# Silencing of *Adc* and *Ebony* Causes Abnormal Darkening of Cuticle in *Henosepilachna vigintioctopunctata*

**DOI:** 10.3389/fphys.2022.829675

**Published:** 2022-02-24

**Authors:** Long-Ji Ze, Lin Jin, Guo-Qing Li

**Affiliations:** Education Ministry Key Laboratory of Integrated Management of Crop Diseases and Pests/State & Local Joint Engineering Research Center of Green Pesticide Invention and Application, Department of Entomology, College of Plant Protection, Nanjing Agricultural University, Nanjing, China

**Keywords:** N-β-alanyldopamine, biosynthesis, adult cuticle, pigmentation, *Henosepilachna vigintioctopunctata*

## Abstract

N-β-alanyldopamine (NBAD) is a precursor of N-acylquinone sclerotin utilized for cross-linking between cuticular proteins for cuticle during insect molting. The importance of NBAD in cuticle tanning has not been well compared among different developing stages of insects. *Henosepilachna vigintioctopunctata*, a typical polyphagous pest feeding on a large number of Solanaceae and Cucurbitaceae plants in Asian countries, displays diverse cuticle pigmentation patterns among developing stages and body regions. Here, we found that the expression of three genes (*Hvadc*, *Hvebony*, and *Hvtan*) involved in NBAD biosynthesis peaked in the 4-day-old pupae or 0-day-old adults of *H. vigintioctopunctata*. At the first, second, third, and fourth larval instar and pupal stage, their transcript levels were high just before and/or right after the molting. Moreover, they were more abundantly transcribed at the larval heads than in the bodies. RNA interference (RNAi) of either *Hvadc* or *Hvebony* at the third instar larvae selectively deepened the color of the larval head capsules, antennae, mouthpart, scoli, strumae, and legs; and depletion of the two genes blackened the pupal head capsules, antennae, mouthpart, and legs. However, the knockdown of either *Hvadc* or *Hvebony* darkened the whole bodies of the adults. Conversely, RNAi of *Hvtan* at the third instar stage had little influence on the pigmentation in the larvae, pupae, and adults. These findings demonstrated that Adc and Ebony are important in cuticle pigmentation of *H. vigintioctopunctata* and suggested that larger quantities of NBAD were present in adults and play more important roles in pigmentation than larvae/pupae.

## Introduction

In insects, the cuticle provides protection against physical injury and water loss, rigidness for muscle attachment and mechanical support, and flexibility in intersegmental and joint areas for mobility. During growth and metamorphosis, insects need to regularly shed off old exoskeletons and synthesize new cuticles to fit the continuously increasing body sizes and the stage-specific body shapes. The newly formed cuticle, mainly composed of cuticular proteins, chitin, and sclerotized reagents, needs to be tanned, a process involved in melanization and sclerotization (Sugumaran and Barek, 2016). Moreover, melanization and sclerotization of insect cuticles also determine pigmentation and play an important role in ecological adaption, such as in escaping predation, mimicry, sexual selection, signaling, and thermoregulation (Wright, 1987; True, 2003; Wittkopp and Beldade, 2009; van’t Hof et al., 2011).

From a biochemical perspective, melanization and sclerotization of insect cuticles result from a combination of dark black and brown melanin and light yellow and colorless sclerotins (Wright, 1987; True, 2003; Wittkopp et al., 2003; Wittkopp and Beldade, 2009; Zhan et al., 2010). These melanizing and sclerotized reagents are produced from tyrosine. The tyrosine hydroxylase (TH) converts tyrosine to dopa. Subsequently, the dopa decarboxylase (DDC) converts dopa into dopamine. Dopa and dopamine are further converted to corresponding melanins (Andersen, 2010). The production of sclerotins from dopamine has been clarified in *Drosophila melanogaster* (Phillips et al., 2005). There are four reaction steps: (1) N-acylation of dopamine with acetyl-CoA to N-acetyldopamine (NADA). (2) Decarboxylation of aspartic acid to β-alanine by aspartate 1-decarboxylase (ADC, Black). (3) N-acylation of dopamine with β-alanine to produce N-β-alanyldopamine (NBAD) by NBAD synthase (Ebony). This reaction is reversible, with the reverse reaction catalyzed by an NBAD hydrolase (Tan). (4) Oxidation of NADA and NBAD to NADA-quinone and NBAD-quinone, which are polymerized to form the corresponding N-acylquinoid sclerotins (Phillips et al., 2005; Simon et al., 2009; Noh et al., 2016; Mun et al., 2020).

The importance of normal pigmentation of two enzymes, ADC and Ebony, has been confirmed (Mun et al., 2020). For ADC, levels of β-alanine are reduced in the heads of *adc* mutants compared to wild type in *D. melanogaster* (Hodgetts and Choi, 1974; Phillips et al., 2005; Borycz et al., 2012; Ziegler et al., 2013), in black body color mutant *Tribolium castaneum* (Kramer et al., 1984) and the black pupal (*bp*) mutant of *Bombyx mori* (Dai et al., 2015). Deficiency of β-alanine causes a lack of NBAD and leads to black body color (Kramer et al., 1984; Dai et al., 2015). Treatment with β-alanine can restore these mutants to wild-type phenotype (Jacobs, 1974; Kramer et al., 1984; Roseland et al., 1987; Wappner et al., 1996a,b; Phillips et al., 2005).

As for Ebony, the mutation in or knockdown of *ebony* increases black pigments in Dipteran insects *D. melanogaster* and *Ceratitis capitata* (Bridges and Morgan, 1923), Lepidopteran insects *B. mori* (Futahashi et al., 2008) and *Spodoptera litura* (Bi et al., 2019), Coleopteran insects *Tenebrio molitor* (Mun et al., 2020) and *T. castaneum* (Tomoyasu et al., 2009), and Hemipteran insect *Oncopeltus fasciatus* (Liu et al., 2016). Similarly, Tan has been recognized as an additional factor that promotes melanization in *Heliconius* (Ferguson et al., 2011). In *D. melanogaster*, loss of Tan causes a global reduction of melanin patterns (True et al., 2005; Jeong et al., 2008). However, the importance of NBAD in cuticle tanning has not been well compared among different developing stages of insects.

*Henosepilachna vigintioctopunctata* (Fabricius) (Coleoptera: Coccinellidae) is a typical polyphagous pest that feeds on a large number of Solanaceae and Cucurbitaceae plants in Asian countries (Zhang et al., 2018). The color markings in the beetle are distinctive and variable in different developing stages (Casari and Teixeira, 2015). This offers a very suitable model to test the pigmentation patterns. Here, using RNA interference (RNAi) technology, the three genes *adc*, *ebony*, or *tan* reported being involved in NBAD biosynthesis were knockdown at the third larval instar stage of *H. vigintioctopunctata*. The cuticle pigmentation patterns were detected and compared during larval–larval, larval–pupal, and pupal–adult molting. The results demonstrate that Adc and Ebony are important in cuticle pigmentation of *H. vigintioctopunctata* and suggest that larger quantities of NBAD were present in adults and play more important roles in pigmentation than larvae/pupae.

## Materials and Methods

### Insect

*Henosepilachna vigintioctopunctata* adults were collected from *Solanum melongena* L. in Nanjing city, Jiangsu Province, China, in the summer of 2018. The beetles were routinely maintained in an insectary at 28 ± 1°C under a 14:10 h light-dark photoperiod and 50–60% relative humidity using potato (*Solanum tuberosum*) foliage at the vegetative growth or young tuber stages to assure sufficient nutrition. Under this feeding protocol, the larvae progressed through four distinct instars, with approximate periods of the first, second, third, and fourth instar stages of 3, 2, 2, and 3 days, respectively. Upon reaching full size, the fourth larval instars stopped feeding, fixed their abdomen ends to the substrate surface, and entered the prepupal stage. The prepupae spent approximately 2 days to pupate. The pupae lasted about 4 days and the adults emerged.

### Molecular Cloning

TRIzol reagent (Invitrogen, New York, NY, United States) was used to extract the total RNA following the protocols of the manufacturer. The NanoDrop 2000 spectrophotometer (Thermo Fisher Scientific, New York, NY, United States) was applied to perform the RNA quantification. RNA purity was determined by assessing optical density (OD) absorbance ratios at OD260/280 and OD260/230. The integrity of RNA was analyzed *via* 1% agarose gel electrophoresis with ethidium bromide staining. Reverse transcription was performed using a PrimeScript™ RT reagent Kit with a gDNA Eraser (TaKaRa Biotechnology Corporation Ltd., Dalian, China). Briefly, the reaction was incubated at 37°C for 15 min and then 85°C for 5 s. The synthesized cDNAs were preserved at −20°C for further use.

The putative *adc*, *ebony*, and *tan* genes were obtained from *H. vigintioctopunctata* transcriptome data (Zhang et al., 2018). The correctness of the sequences was substantiated by PCR using primers in Supplementary Table 1. The sequenced cDNAs were submitted to GenBank (accession numbers: *adc*, MW380963; *ebony*, MW380964; and *tan*, MW380968).

The protein sequences of ADC, Ebony, and Tan from other species were acquired from the NCBI.^1^ Phylogenetic analysis was conducted using MEGA-X software^2^ and the neighbor-joining method with 1,000 bootstrap replications.

### Synthesis of dsRNA Molecules

The cDNA fragments derived from *adc*, *ebony*, *tan*, and enhanced green fluorescent protein (*egfp*) were, respectively, amplified by PCR using specific primers (Supplementary Table 1) conjugated with the T7 RNA polymerase promoter. These targeted regions were further BLAST (BLASTN) searched against *H. vigintioctopunctata* transcriptome data (Zhang et al., 2018) to identify any possible off-target sequences that had an identical match of 20 bp or more. The dsRNA was synthesized using the MEGAscript T7 High Yield Transcription Kit (Ambion, Austin, TX, United States) according to the instructions of the manufacturer. Subsequently, the synthesized dsRNA (at a concentration of 5–8 μg/μl) was determined by agarose gel electrophoresis and the NanoDrop 1,000 spectrophotometer (Thermo Fisher Scientific, Waltham, MA, United States) (data not shown) and kept at −80°C until used in the subsequent experiment.

### Larval RNA Interference

RNA interference of larvae was performed according to a previously described method (Xu et al., 2020; Ze et al., 2021). Briefly, an aliquot (0.1 μl) of the solution including 300 ng dsRNA was injected into the newly ecdysed third instar larvae. Blank and negative control larvae were injected with the same volume of PBS and ds*egfp* solutions, respectively. A group of 15 injected larvae was set as a replicate. Each dsRNA injection was repeated 8 times. Three replicates (each replicate contained at least six individuals) were sampled at 48 and 72 h after injection for qRT-PCR to test RNAi efficacy. Another two replicates were used to observe the phenotype.

Three biological independent experiments were carried out using the newly ecdysed third instar larvae and were planned to determine the RNAi effects of *Hvadc*, *Hvebony*, and *Hvtan* on the performances and cuticle tanning of the resultant larvae, pupae, and adults. Three treatments were set as follows: phosphate-buffered saline (PBS), 300 ng ds*egfp* and 300 ng ds*adc*; PBS, 300 ng ds*egfp* and 300 ng ds*ebony*; or PBS, 300 ng ds*egfp* and 300 ng ds*tan*.

### Real-Time Quantitative PCR

For temporal expression analysis, cDNA templates were derived from 3-day-old eggs, the first, second, third, and fourth larval instar, the prepupae, pupae, and adults at an interval of 1 day (D0 indicates newly molted larvae, pupae, or newly emerged adults). For comparison of the expression levels in different portions, the heads and the remaining portions (bodies) were separately collected from 2-day-old third instar larvae, and 1-, 2-, and 3-day-old fourth instar larvae. Each sample contained 10 individuals and repeated three times. For analysis of the effects of treatments, total RNA was extracted from treated larvae. Each sample contained at least six individuals and repeated three times. The RNA was extracted using SV Total RNA Isolation System Kit (Promega, Madison, WI, United States). cDNA was prepared according to the instructions by using HiScript III RT SuperMix for qPCR (+gDNA wiper) kit (Vazyme, Nanjing, China). The qPCR performed on Applied Biosystems 7500 System (Life Technologies, Carlsbad, CA, United States) with ChamQ Universal SYBR qPCR Master Mix (Vazyme, Nanjing, China) according to the instructions of the manufacturer. Each 20 μl reaction solution containing 10 μl of 2 × ChamQ Universal SYBR qPCR Master Mix, 0.4 μl of each primer (10 μM), 1 μl of cDNA template, and 8.2 μl of nuclease-free water. The cycling parameters were: 1 cycle of 95°C for 3 min; 40 cycles of 95°C and 60°C for 10 s and 30 s, respectively. Quantitative mRNA measurements were performed by qRT-PCR in technical triplicate, using two internal control genes (*ribosomal protein S18*, *HvRPS18*; *ribosomal protein L13*, *HvRPL13*; and the primers listed in Supplementary Table 1) according to the published results (Lü et al., 2018). An RT negative control (without reverse transcriptase) and a non-template negative control were included for each primer set to confirm the absence of genome DNA and to check for primer-dimer or contamination in the reactions, respectively.

According to a previously described method (Bustin et al., 2009), the generation of specific PCR products was confirmed by gel electrophoresis. The primer pair for each gene was tested with a 10-fold logarithmic dilution of a cDNA mixture to generate a linear standard curve [crossing point (CP) plotted vs. log of template concentration], which was used to calculate the primer pair efficiency. All primer pairs amplified a single PCR product with the expected sizes, showed a slope less than −3.0, and exhibited efficiency values ranging from 2.5 to 2.6. Data were analyzed by the 2^–ΔΔ*CT*^ method, using the geometric mean of the four internal control genes for normalization.

### Image Processing and Color Intensity Measurement

Digital photographs of all the phenotypes were taken with Nikon SMZ 25 stereo microscopy (Nikon, Japan). Images of hindwings, dissected from 7-day-old adults whose third instar larvae were treated with dsRNA, were transferred to RGB stack images, and color intensity in equivalent regions was determined as mean grayscale values (average luminance) *via* ImageJ software. Under this measurement, the lower color intensity indicates the darker cuticle pigmentation (Noh et al., 2016). All the treated groups and the corresponding control groups were photographed under the same conditions.

### Data Analysis

Statistical significance of differences in the mRNA expression or other biological experiments among PBS, ds*egfp*, and dsRNA treatment groups was determined by one-way ANOVA and Turkey’s test in SPSS Statistics 20.0 software (at *P* < 0.05). In the color intensity measurement of hindwings, the statistical significance of differences in mean gray values between the dsRNA-treated groups was assessed by Student’s *t*-test.

## Results

### Temporal Expression Pattern of *Adc*, *Ebony*, and *Tan* Transcript

By mining of *H. vigintioctopunctata* transcriptome data and constructing the phylogenetic trees, *Hvadc*, *Hvebony*, and *Hvtan* were identified (Supplementary Figures 1–3).

**FIGURE 1:**
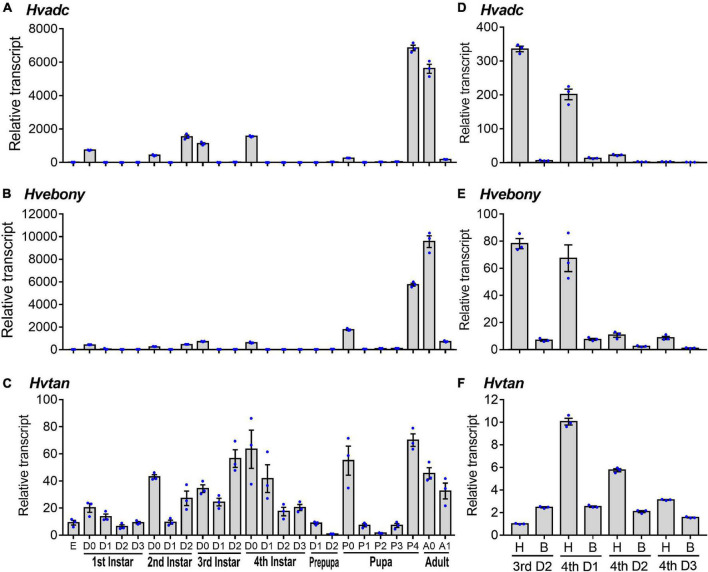
Transcription patterns of *adc*, *ebony*, and *tan* genes in *Henosepilachna vigintioctopunctata*. For temporal expression analysis **(A–C)**, RNA templates were derived from day 3 eggs, the larvae from the first through the fourth instar, prepupae, pupae, and adults (D0 indicated newly ecdysed larvae or pupae, or newly emerged adults). Each repeat included 20–30 individuals and there were three independent pools. For analysis of the expression levels in different regions **(D–F)**, the heads and the remaining bodies were separately collected from the 2-day-old third instar larvae, and 1-, 2- and 3-day-old fourth instar larvae. A replicate included 10 individuals and each sample repeated 3 times. All the samples were measured in technical triplicate using qRT-PCR. The values were calculated using the 2^–ΔΔ*CT*^ method. The lowest transcript level of each of the three mRNAs at a specific developing time is set as 1. The relative transcripts are the ratios of copy numbers in different developing stages relative to larvae at the specific developing time. The genes for *ribosomal protein S18* (*HvRPS18*) and *ribosomal protein L13* (*HvRPS13*) were used as internal controls. The columns represent averages with vertical lines indicating ± SE. E, egg; H, head; B, remaining body without head.

To detect the expression patterns of *Hvadc*, *Hvebony*, and *Hvtan* during different developmental stages, qRT-PCR was performed. The results revealed that *Hvadc*, *Hvebony*, and *Hvtan* were detectable from the embryo (egg) to adults. They peaked in the 4-day-old pupae or 0-day-old adults. Moreover, at the first, second, third, and fourth larval instar and pupal stages, the transcription levels of *Hvadc*, *Hvebony*, and *Hvtan* were high just before and/or right after the molt, and were low at the intermediate stages (Figures 1A–C).

The expression levels of *Hvadc*, *Hvebony*, and *Hvtan* in larval heads were compared to those in the bodies of 2-day-old third instar larvae, and 1-, 2-, and 3-day-old fourth instar larvae. Both *Hvadc* and *Hvebony* were abundantly transcribed in the larval heads of 2-day-old third instar larvae and 1-day-old four instar larvae (Figures 1D,E). Similarly, the levels of *Hvtan* in the head samples of 1- to 3-day-old fourth instar larvae were higher than those in the bodies (Figure 1F).

### RNA Interference of *Adc* Blackens the Larvae

**FIGURE 2:**
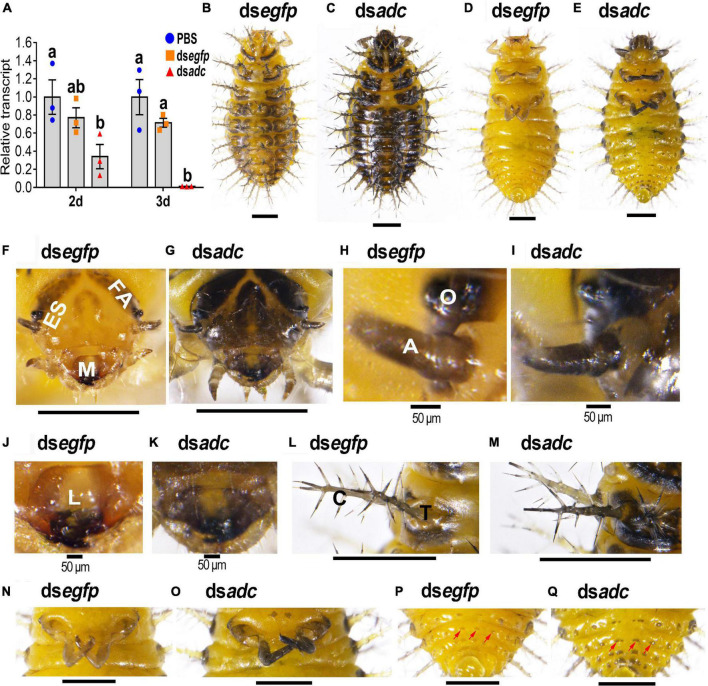
RNA interference of *Hvadc* darkens larvae of *H. vigintioctopunctata*. The newly ecdysed third instar larvae were injected with an aliquot (0.1 μl) of PBS, or solution including 300 ng ds*egfp* or 300 ng ds*adc*. The larvae were then transferred to potato foliage. Two days and three days after injection, the expression level of *Hvadc* was measured **(A)**. Relative transcripts are the ratios of relative copy numbers in treated individuals to that in PBS-injected ones, which is set as 1. Different letters indicate significant differences at *P*-value < 0.05 using ANOVA with the Tukey–Kramer test. The dorsal and ventral views of ds*egfp*- **(B,D)** and ds*adc*- **(C,E)** treated larvae are shown. The heads **(F,G)**, ocelli and antennae **(H,I)**, mouthpart **(J,K)**, scoli and strumae **(L,M)**, hind legs **(N,O)**, and the end of abdomens **(P,Q)** were further amplified. ES, epicranial suture; FA, frontal arms; M, mouthpart; O, ocelli; A, antennae; C, scolus; T, struma. Red arrows point to mastoids on the abdomen.

Three days after the introduction of ds*adc* into the newly ecdysed third instar larvae, the accumulated mRNA level of the target transcript was heavily suppressed (Figure 2A).

All the controls (PBS- and ds*egfp*-introduced) and ds*adc*-treated larvae normally molted to the fourth instar larvae. However, the color was darkened in the heads of the *Hvadc* RNAi larvae, compared with those of the PBS- and ds*egfp*-treated ones (Figures 2B,D vs. C,E), especially the mouthparts (Figures 2F vs. G) and the patches around larval ocelli and the antennae (Figures 2H vs. I). In addition, a wide cream band called epicranial suture (ES) was narrowed, and the V-shaped frontal arms and the U-shaped patch on the head top were widened in the *Hvadc* RNAi larvae (Figures 2F vs. G).

In the mouthparts of the control fourth instar larvae (PBS- and ds*egfp*-injected), the median part of the labrum is cream, while the lateral and anterior parts are brownish narrow bands. Other appendages of the mouthparts, namely, mandibles, maxilla, and labium were black in color (Figures 2F,J). By contrast, in the *Hvadc* RNAi larvae, the color of all the pigmented regions was deepened and blackened (Figures 2F,J vs. G,K). Moreover, the scoli, strumae, and legs were all darker-colored in the *Hvadc* silenced larvae (Figures 2L,N vs. M,O). In addition, small mastoids queuing up on the abdomen were darker pigmented in ds*adc*-injected larvae, compared with that of ds*egfp*-injected larvae (Figures 2D,P vs. E,Q).

### Silencing *Adc* Affects Coloration of Pupae

**FIGURE 3:**
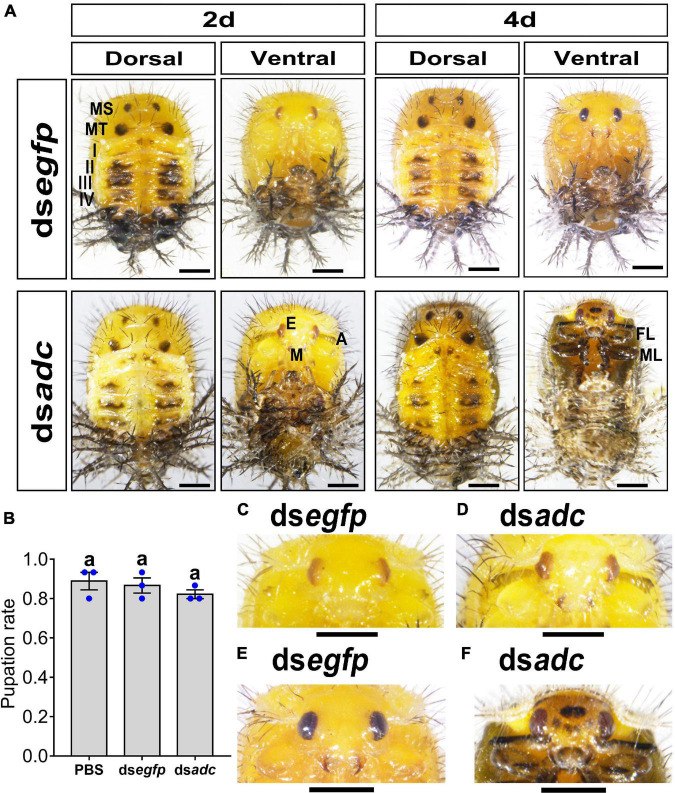
Knockdown of *Hvadc* darkens pupa color in *H. vigintioctopunctata*. The newly molted third instar larvae were injected with an aliquot (0.1 μl) of PBS, or solution including 300 ng ds*egfp* or 300 ng ds*adc*. The larvae were then transferred to potato foliage. The pupation rate was recorded during a 3-week trial period **(B)**. The averages (±SE) following different letters indicate significant differences at *P*-value < 0.05 using ANOVA with the Tukey–Kramer test. The dorsal and ventral views of 2-day-old and 4-day-old pupae whose third instar larvae had been subjected to ds*egfp* and ds*adc* injection are shown **(A)**. The heads of 2-day-old pupae **(C,D)** and 4-day-old pupae **(E,F)** were further amplified. MS, meso thorax; MT, meta thorax; I–IV, tergites I–IV; E, compound eye; A, antenna; M, mouthpart; FL, foreleg; ML, midleg. Scale bars: 1 mm.

Knockdown of *Hvadc* did not affect the pupal morphology (Figure 3A) and the pupation rate (Figure 3B), but the coloration.

The integument of the 2-day-old pupae developed from the third instar larvae treated with ds*egfp* or ds*adc* were generally pale yellow, especially on the ventral one (Figure 3A, left panel). Then, the overall color shifted to dull yellow in the 4-day-old pupae (Figure 3A, right panel). From the dorsal views, the paired black markings in the meso and meta thoraxes, and the dark patches in tergite I–IV were similar between the control (ds*egfp*-treated) and the *Hvadc* RNAi pupae (Figure 3A).

However, the antennae and mouthparts of the 2-day-old *Hvadc*-silenced pupae were blackened (Figure 3D), while those parts in 2-day-old pupae treated with ds*egfp* exhibited no obvious pigmentation (Figure 3C). Four days after pupation, the mouthparts of control displayed rufous color, and the color of antennae and legs did not obviously change (Figure 3E). By contrast, the cuticle color between two compound eyes of the *Hvadc-*silenced pupae was obviously darker, and obvious central black patches and several small dark spots were present (Figures 3E vs. F). The antennae, mouthparts, and legs became darker (Figure 3F).

### Knockdown of *Adc* Affects the Color of Adult Cuticle and Wings

**FIGURE 4:**
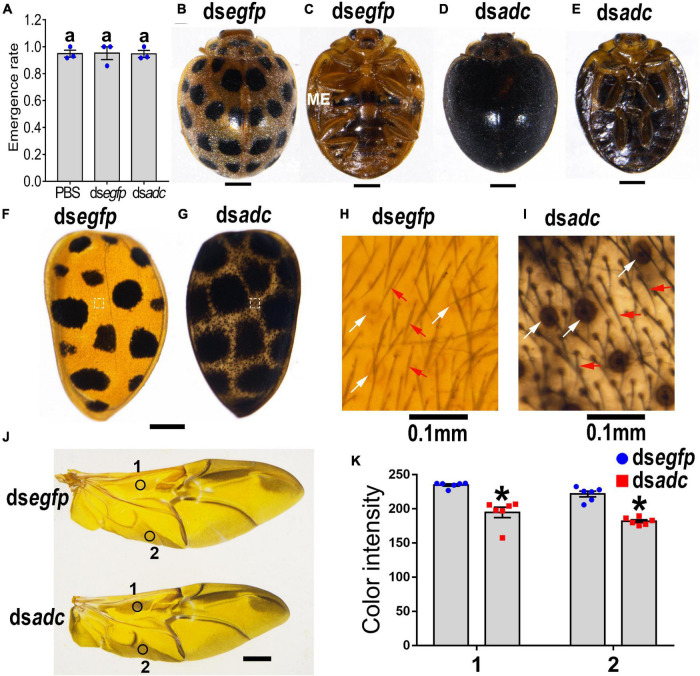
Silencing *Hvadc* affects pigmentation in adults of *H. vigintioctopunctata*. The newly molted third instar larvae were injected with an aliquot (0.1 μl) of PBS, or solution including 300 ng ds*egfp* or 300 ng ds*adc*. The larvae were then transferred to potato foliage. The emergence rates and the rates of normal adults were recorded during a 3-week trial period **(A)**. The average values (±SE) followed by different letters indicate significant differences at *P*-value < 0.05 using ANOVA with the Tukey–Kramer test. The dorsal **(B,D)** and ventral **(C,E)** views of 7-day-old adults whose third instar larvae had been subjected to ds*egfp* or ds*adc* treatment are shown. The elytra **(F,G)** and the elytral surfaces were further amplified **(H,I)**. The hindwings **(J)** and the color intensity **(K)** in equivalent regions of them (circles 1 and 2 in **J**) were determined as mean gray values (average luminance) by ImageJ software. Data are shown as the mean values ± SE (*n* = 6). The asterisk indicates a significant difference in color intensity between control and test beetles (*P* < 0.01, *t*-test). White and red arrows point to the circle pits and setae, respectively. ME, metathorax.

The emergence rate of the pupae in the ds*adc*-treated group was 95% on average, showing no significant difference with the one in the PBS- or ds*egfp*-treated group (Figure 4A). For *Hvadc* RNAi adults, 28 black markings were distributed symmetrically on two hard elytra 1 day after emergence. Then, the black pigments were gradually deposited and finally covered the whole body from the dorsal view 7 days after emergence, while the pigmentation of the control (ds*egfp*-treated) adult remained the same (Figures 4B vs. D). Similarly, a pair of black markings on the sternum of the metathorax was seen in the control and 1-day-old *Hvadc* RNAi adults; while the whole body from the ventral view was blackened in the 7-day-old *Hvadc* RNAi adults (Figures 4C vs. E). Knockdown of *Hvadc* caused the overall darkening pigmentation in elytra, which seems to be due to the enlargement of the black spots (Figures 4F vs. G). Moreover, the view of the amplified elytra from *Hvadc* RNAi adults showed that the setae and circle pits were all dark-colored, in contrast to the reddish-brown color in the control beetles (Figures 4H vs. I). Further dissection of the head, pronotum, scutellum, hindlegs, and hindwing revealed that these regions all shifted to the darker pigmentation (Figure 4J and Supplementary Figure 4). The color intensity of the corresponding regions of hindwings showed a significant difference between ds*adc* and ds*egfp* treated groups (Figure 4K).

### RNA Interference of *Ebony* on the Third Instar Larvae

**FIGURE 5:**
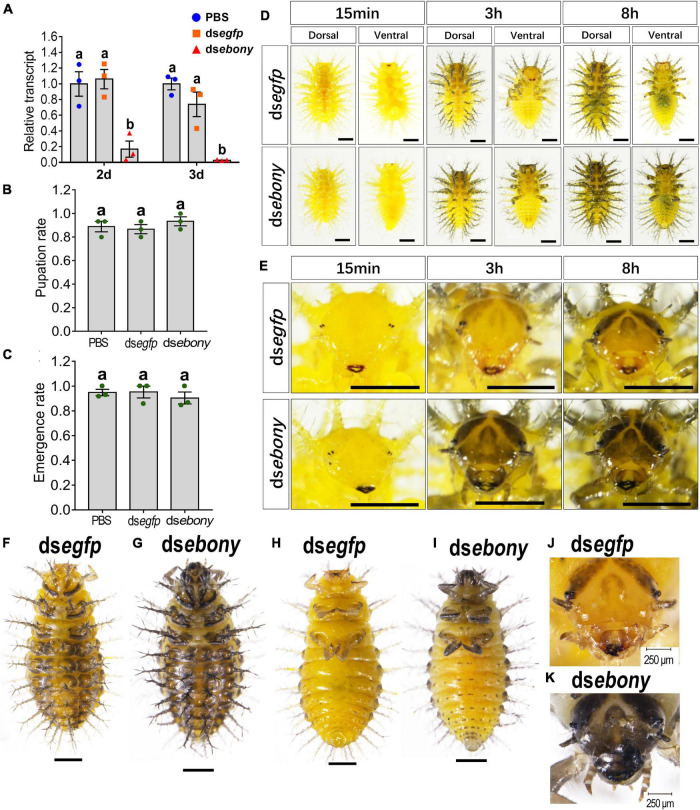
Depletion of *Hvebony* influences pigmentation in larvae of *H. vigintioctopunctata*. The newly molted third instar larvae were injected with an aliquot (0.1 μl) of PBS, or solution including 300 ng ds*egfp* or 300 ng ds*ebony*. The larvae were then transferred to potato foliage. Two days and three days after injection, the expression level of *Hvebony* was measured **(A)**. Relative transcripts are the ratios of relative copy numbers in treated individuals to that of PBS-treated ones, which is set as 1. The pupation and emergence rates were recorded during a 3-week trial period **(B,C)**. Different letters indicate significant difference at *P-*value < 0.05 using ANOVA with the Tukey–Kramer test. The dorsal and ventral views of ds*egfp*- and ds*ebony*-treated larvae **(D)** and amplified larval heads **(E)** at different times after molting to the fourth instar, and the dorsal and ventral views of larvae in the wandering stage **(F–I)** are shown. The heads of the wandering stage larvae were further amplified **(J,K)**. The unlabeled scale bars: 1 mm.

Since both the Adc and Ebony play central roles in the synthesis of the NBAD (Massey et al., 2019), we have knocked down the expression of *Hvebony* by injection of ds*ebony* to the newly ecdysed third instar larvae (Figure 5A).

The pupation rate and the emergence rate of the *Hvebony* RNAi larvae were similar to those of the control larvae which were injected PBS or ds*egfp* (Figures 5B,C). The mandibles were darker-colored in the ds*ebony*-introduced newly molted fourth instar larvae, compared with those in the control larvae treated with PBS or ds*egfp* (Figures 5D,E, 15 min). Eight hours postmolting, the scoli and strumae from the larval thorax to the eighth segment of the abdomen, along with head and leg, became brownish in the PBS- and ds*egfp*-introduced larvae (Figures 5D,E, above panel). By contrast, the color of the body parts, namely, head, scoli, strumae, and legs became dark brown in the ds*ebony*-treated larvae (Figures 5D,E, lower panels).

Similarly, at the wandering stage, the cuticles of the larvae head capsules, scoli, strumae, legs, and spots on the abdomen were more blackened in *Hvebony* RNAi larvae than those in the control larvae (Figures 5F,H,J vs. G,I,K).

**FIGURE 6:**
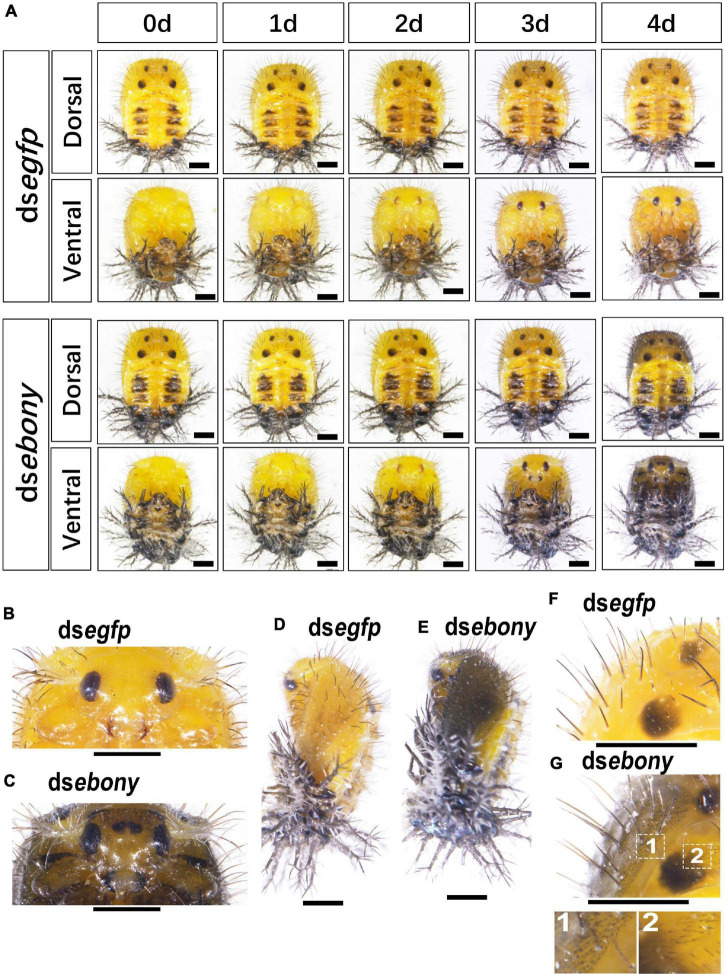
RNA interference of *Hvebony* affects the pigmentation in pupae of *H. vigintioctopunctata*. The dorsal and ventral views of 0-day, 1-day, 2-day, 3-day, and 4-day old pupae from the third instar larvae injected with ds*egfp* and dse*bony* **(A)** are shown. The heads **(B,C)** and the dorsal view **(F,G)** of the 4-day-old pupae were further amplified. The lateral views of 4-day-old pupae **(D,E)** are shown. The unlabeled scale bars: 1 mm.

In the ds*ebony*-introduced pupae, the black dorsal markings in the meso and meta thoraxes and the dark patches in tergite I–IV showed no significant difference with PBS or ds*egfp*-treated pupae (Figure 6A). However, the mandibles were darker in the 3-day/4-day-old *Hvebony*-silenced pupae, than those in control groups (Figure 6A). Similar to the phenotype of the *Hvadc* depleted pupae, several small dark spots appeared in the cuticle between two compound eyes of the ds*ebony*-introduced 4-day-old pupae (Figures 6B vs. C). Furthermore, the cuticle of the head, legs, elytra, and pronotum in ds*ebony*-introduced 4-day-old pupae became significantly darker than that of pupae injected with PBS or ds*egfp* (Figures 6B,D,F vs. C,E,G). The enlarged dorsal view displayed that many short black setae and dots appeared on the cuticle of the ds*ebony*-injected 4-day-old pupae to make the cuticle blacker in color than the control (Figures 6F vs. G).

**FIGURE 7:**
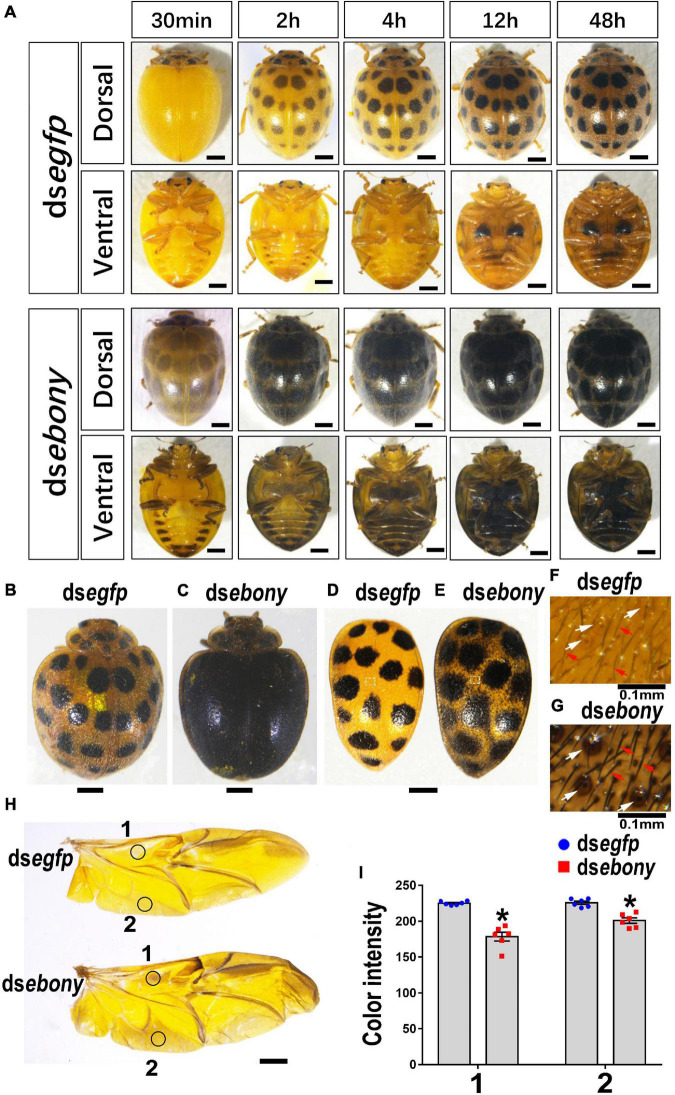
Depletion of *Hvebony* deepens color of adults in *H. vigintioctopunctata*. The dorsal and ventral views of adults from different times after emergence **(A)**. The 7-day-old adults **(B,C)** and elytra dissected from 7-day-old adults **(D,E)** are shown. The surfaces of elytra were further amplified **(F,G)**. The hindwings **(H)** and the color intensity **(I)** in equivalent regions of them (circles 1 and 2 in **H**) were determined as mean gray values (average luminance) by ImageJ software. Data are shown as the mean values ± SE (*n* = 6). The asterisk indicates a significant difference in color intensity between control and test beetles (*P* < 0.01, *t*-test). The unlabeled scale bars: 1 mm. White and red arrows point to the pits and setae, respectively.

Thirty minutes after emergence, the elytra of the control adults treated with PBS or ds*egfp* still exhibited light yellow in color. Subsequently, the elytra turned copper yellow, and 28 spots appeared, gradually darkened (Figure 7A, ds*egfp*-dorsal view). By contrast, knockdown of *Hvebony* led to an overall gray pigmentation and enlarged 28 dark spots in elytra within 30 min after emergence. The elytra of the *Hvebony* RNAi adults also gradually darkened (Figure 7A, ds*ebony*-dorsal view), but they looked completely black in color 7 days after emergence (Figures 7B vs. C). Further dissection of the head, pronotum, scutellum, and hindlegs of the *Hvebony* RNAi adults showed that these regions all turned to darker pigmentation than control adults (Supplementary Figure 4). Similar to the *Hvadc*-silenced adults, the 28 black spots on the elytra of the *Hvebony*-silenced adults became larger than those on the control elytra (Figures 7D vs. E), and the color of the setae and pits on the surface of the elytra was darker than that of the control elytra (Figures 7F vs. G). In addition, the pigment regions of the hindwing from the *Hvebony* RNAi adults were darker than controls (Figures 7H,I).

From the ventral view, six pairs of dark patches appeared along the lateral portions on the abdomen segments of the *Hvebony* RNAi adults, and the last two pairs extended and merged together. The pale-yellow color outside the dark patches was gradually darkened, until merging the dark patches 2 days after emergence (Figure 7A, ds*ebony*-ventral view). The legs and the abdomen of the *Hvebony*-depleted adults were blacker in color than the control beetles (Figure 7A, ds*ebony*-ventral view).

### RNA Interference of *Tan* on the Third Instar Larvae

Two days and three days after injection of ds*tan* to the third instar larvae, the expression of the target *Hvtan* transcript was significantly reduced, compared with the control larvae injected PBS or ds*egfp* (Supplementary Figure 5A).

Depletion of *Hvtan* exerted little influence on pupation and emergence rates (Supplementary Figure 7). The results of color intensity measurement (data not shown) indicated that knockdown of *Hvtan* had a very limited influence on the cuticle pigmentation at the larval, pupal, and adult stages, compared with the controls subjected to PBS or ds*egfp* injection (Supplementary Figures 5B,C, 6A–M). In addition, there was no significant difference in the color intensity of the hindwings between ds*egfp*- and ds*tan*-treated ladybirds (Supplementary Figure 6N).

## Discussion

The conjugation and cross-linking of cuticle proteins during cuticle tanning lead to an insoluble, hard, and darkened red-brown exoskeleton in Coleoptera (Roseland et al., 1987). NBAD, a sclerotic reagent, and a pigment precursor play critical roles in cuticle pigmentation and hardening (Kramer et al., 1984). In this article, three genes (*Hvadc*, *Hvebony*, and *Hvtan*) reported to be involved in NBAD biosynthesis were identified and their role in the pigmentation of cuticle on the larvae, pupae, and adults of *H. vigintioctopunctata* was explored by RNAi in the newly ecdysed third instar larvae. Three pieces of experimental evidence suggested that NBAD plays more important roles in the pigmentation in adults than that in the larvae and pupae of *H. vigintioctopunctata*.

First, we found that *Hvadc*, *Hvebony*, and *Hvtan* were detected from the embryo (egg) to adults. Moreover, the transcription levels of *Hvadc*, *Hvebony*, and *Hvtan* were high just before and/or right after the molt at the first, second, third, and fourth larval instar and pupal stages (Figure 1). In *S. litura*, *Slebony* was expressed at all developing stages, namely, egg, the first through sixth larval instar larvae, prepupa, pupa, and adult (Bi et al., 2019). Similarly, *ebony* is abundantly expressed at the larval, pupal, and adult stages in *P*apilio *xuthus* and *Papilio machaon* (Li et al., 2015). In *T. molitor*, the abundant transcripts were confirmed at late stages of development from pharate pupae through 10-day-old adults (Mun et al., 2020). The expression profiles of *Hvadc*, *Hvebony*, and *Hvtan* suggest that the three functional enzymes catalyze the formation of melanin and sclerotins after each molting during the developing stages.

Second, the expression of the *Hvadc*, *Hvebony*, and *Hvtan* peaked in the 4-day-old pupae or 0-day-old *H. vigintioctopunctata* adults (Figure 1). The highest expression levels at the late pupal and early adult phrases suggest that abundant NBAD were produced in the preadults and adults. In agreement with these results, the highest level of *Tmadc* is seen right after adult emergence (adult day 0) in *T. molitor* (Mun et al., 2020). Similarly, high levels of *Slebony* were found just before and right after adult emergence in *S. litura* (Bi et al., 2019). Moreover, *Dmadc* was expressed at high levels at the end of the pupae period (96 h after pupation) in *D. melanogaster* (Sobala and Adler, 2016).

Lastly, the results revealed that RNAi of either *Hvadc* or *Hvebony* selectively deepened the color of the larval head capsules, scoli, strumae, and legs (Figures 2, 5), and depletion of *Hvadc* or *Hvebony* blackened the pupal head capsules, antennae, mouthpart, and legs (Figures 3, 6), while knockdown of either *Hvadc* or *Hvebony* darkened whole bodies of the adults (Figures 4, 7) of *H. vigintioctopunctata*. The greater accumulation of black pigments in the *Hvadc* or *Hvebony* depleted adults likely resulted from the conversion of larger quantities of dopamine to brown dopamine-melanin than in the larvae. Therefore, we infer that larger quantities of NBAD were present in adults than larvae/pupae.

 In contrast, microinjection of ds*adc* into penultimate-instar larvae does not cause obvious phenotype in the *T. castaneum* old larvae but leads to a darkened body pigmentation in pupae and adults when compared with controls (Arakane et al., 2009). In the *Bmadc* mutant strain of *B. mori*, β-alanine and NBAD were deficient but dopamine is accumulated. As a result, deep coloration was induced in pupae (Dai et al., 2015). It appears that NBAD is as important in pupae as that in adults in both *T. castaneum* (Arakane et al., 2009) and *B. mori* (Dai et al., 2015).

As for *ebony*, CRISPR/Cas9-mediated *ebony* knockout did not change the pigmentation in larvae but led to darker coloration in the pupae and adults in *S. litura* (Bi et al., 2019). In *D. melanogaster*, homozygous mutations of *ebony* bring about dark body pigmentation in adult flies, but not larvae and pupae (Massey et al., 2019). Moreover, the core enhancers in the *ebony* gene determine thoracic pigmentation intensity in different *D. melanogaster* strains across geographical gradients (Telonis-Scott et al., 2011; Telonis-Scott and Hoffmann, 2018). These results suggest that reduced synthesis of NBAD leads to abnormal accumulations of high levels of dopamine, which likely undergoes dopamine-melanin synthesis to cause a dark coloration of cuticle in adults of insects from different orders. Interestingly, although the elytra of adults treated with ds*adc* and ds*ebony* appear to be almost completely black in color, we found that the main regions of elytra got dark pigmented were pits and setae (Figures 4, 7). Similarly, as early as the end of the pupae, the epidermal setae were already black in coloration in the *Hvebony* depleted pupae (Figure 6). It suggested that NBAD may be mainly accumulated in pits and setae of *H. vigintioctopunctata* cuticle.

It is known that NBAD is a major sclerotizing precursor that is secreted during the cuticle tanning process. It is further oxidized and used to cross-link cuticular proteins to form a rigid coleopteran exoskeleton at the adult stage (Noh et al., 2016). Moreover, some pigmentation gene transcripts, such as *ebony* and *tan*, are pleiotropic genes that affect more than one trait. In *D. melanogaster*, loss-of-function mutations in *ebony* and *tan* also affect cuticular hydrocarbon composition (Massey et al., 2019). A bias for long-chain hydrocarbon increases the melting temperatures and enables the flies to live longer in dry climates (Gibbs, 1998). *H. vigintioctopunctata* is a diurnal herbivorous ladybird. The adults are frequently exposed to a dry environment to forage for food, oviposition sites, and copulation mates. Hard exoskeleton in the adults confers greater tolerance to desiccation and ultraviolet during the foraging stage (Matute and Harris, 2013; Bastide et al., 2014).

In addition, we noticed that the change of cuticle pigmentation was very limited in the beetles treated with ds*tan* (Supplementary Figures 5, 6). The enzyme encoded by *tan* is required for the production of dopamine in *D. melanogaster* (True et al., 2005). In *B. mori*, *tan* is suggested to be the responsible gene for larval color mutant *rouge*, and Tan plays a significant role in emphasizing the black markings of the larvae (Futahashi et al., 2010). Previous studies have shown that Tan catalyzes the production of dopamine from NBAD during pigment development (True et al., 2005). This role of Tan seems to be not obvious in *H. vigintioctopunctata*. Combined with the results of RNAi of *Hvadc* and *Hvebony*, we speculated that *Hvtan* plays a weak role in the process of converting NBAD to dopamine in *H. vigintioctopunctata*.

## Conclusion

In conclusion, the study has determined the important role of Adc and Ebony in cuticle pigmentation in the 28-spotted ladybird. Larger quantities of NBAD were suggested to be present in adults and play more important roles in pigmentation than larvae/pupae. Abundant NBAD in the adults may be an adaptation strategy in the tanning process to form inconspicuous pigmentation in *H. vigintioctopunctata* beetles.

## Data Availability Statement

The datasets presented in this study can be found in online repositories. The names of the repository/repositories and accession number(s) can be found in the article/Supplementary Material.

## Author Contributions

L-JZ, LJ, and G-QL conceived the study, participated in the design of the experiments and the interpretation of the results, and wrote the first draft of the manuscript. L-JZ and LJ performed the experiments. LJ and G-QL revised the manuscript. All authors contributed to the article and approved the submitted version of the manuscript.

## Conflict of Interest

The authors declare that the research was conducted in the absence of any commercial or financial relationships that could be construed as a potential conflict of interest.

## Publisher’s Note

All claims expressed in this article are solely those of the authors and do not necessarily represent those of their affiliated organizations, or those of the publisher, the editors and the reviewers. Any product that may be evaluated in this article, or claim that may be made by its manufacturer, is not guaranteed or endorsed by the publisher.

## Footnotes

1
https://www.ncbi.nlm.nih.gov/

2
https://sourceforge.net/projects/mega5/
