# Supplementary material for: Silencing of Adc and Ebony Causes Abnormal Darkening of Cuticle in Henosepilachna vigintioctopunctata
Source: Front Physiol. 2022 Feb 24;13:829675. doi: 10.3389/fphys.2022.829675 (PMC8907826; doi:10.3389/fphys.2022.829675)
Supplement: Supplementary file 1 [file Data_Sheet_1.ZIP › supplementary data.doc]

Supplementary data

**Silencing of *Adc* and *Ebony* causes abnormal darkening of cuticle** **in *Henosepilachna vigintioctopunctata***

Long-Ji Ze, Lin Jin*, Guo-Qing Li

Education Ministry Key Laboratory of Integrated Management of Crop Diseases and Pests/ State & Local Joint Engineering Research Center of Green Pesticide Invention and Application, Department of Entomology, College of Plant Protection, Nanjing Agricultural University, Nanjing 210095, China

**Table S1. Primers used in RT-PCR, dsRNA synthesis and qRT-PCR**

| **Fragment name** | **Forward primer** | **Reverse primer** | **Product length(bp)** |
| --- | --- | --- | --- |
| **RT-PCR** |  |  |  |
| *Hvadc* | TACACAAATGATGCTCTGC | TAATCCAAGCGTTTACACT | 1193 |
| *Hvebony* | TGATTGTGGAGGATTTATCGA | GGTAACTTCACTGAATTAGTTGAAC | 2678 |
| *Hvtan* | CTGCTACTCACAAACTGCG | GGCGACTAGGTATAGGAAA | 1256 |
| **dsRNA synthesis** |  |  |  |
| ds*adc* | *TAATACGACTCACTATAGGG*AAAGAAAAGCAGAGCACTGTTGAG | *TAATACGACTCACTATAGGG*AAAATGGACATCCTGGATTTAGAAG | 341 |
| ds*ebony* | *TAATACGACTCACTATAGGG*CGCATTCGCAAAGCTGTATCG | *TAATACGACTCACTATAGGG*GGTCATCGTTGTTATTGTTCGTGTT | 435 |
| ds*tan* | *TAATACGACTCACTATAGGG*TTGATGTGGGAAGAACTTTCGG | *TAATACGACTCACTATAGGG*GGATTGCCTGATTGTTTGTATTGAC | 358 |
| ds*egfp* | *TAATACGACTCACTATAGGG*AAGTTCAGCGTGTCCG | *TAATACGACTCACTATAGGG*CACCTTGATGCCGTTC | 454 |
| **qRT-PCR** |  |  |  |
| *qHvadc* | ACTGAATATGCTTGTCTCCT | CCTCAATGCTGCCTATCT | 77 |
| *qHvebony* | GTGCCCATAGGTCTACCAGT | CAAATTAAGGCCAGCCACGA | 111 |
| *qHvtan* | TCAGAGACTCAGGAGTTGGC | TGACGTGCATGAAGTGTTCC | 171 |
| *qHvRPS18* | CGCAATCAAAGGTGTTGGAAG | GCCTAGGGTTGGCCATAATAG | 134 |
| *qHvRPL13* | AGCATCCTTCGCTCGTTTAG | TTCGACAACCTGCCATTAGG | 137 |

**Figure S1. Phylogenetic analysis of** **aspartate 1-decarboxylases (ADCs) proteins.** The ADC-like sequences originated from Lepidopteran species *Spodoptera frugiperda*, *Spodoptera litura*, *Helicoverpa armigera*, *Bombyx mori and Manduca sexta*; Blattaria species *Zootermopsis nevadensis*; Coleopteran species *Henosepilachna vigintioctopunctata*, *Tribolium castaneum*, *Tenebrio molitor* and *Diabrotica virgifera virgifera*; Hemiptera species *Nilaparvata lugens*; and Diptera species *Drosophila melanogaster*. The tree is constructed using the neighbor-joining method based on the full-length protein sequence alignments. Bootstrap analyses of 1000 replications are carried out and bootstrap values > 50% are shown on the tree.

**Figure S2. Phylogenetic analysis of β-alanyl dopamine** **synthase Ebony proteins.** The Ebony-like sequences originated from Hemiptera species *Nilaparvata lugens* and *Oncopeltus fasciatus*; Diptera species *Drosophila melanogaster* and *Aedes aegypti*; Lepidopteran species *Spodoptera frugiperda*, *Helicoverpa armigera*, *Bombyx mori*, *Manduca sexta* and *Chilo suppressalis*; and Coleopteran species *Henosepilachna vigintioctopunctata*, *Harmonia axyridis*, *Tribolium castaneum* and *Tenebrio molitor*. The tree is constructed using the neighbor-joining method based on the full-length protein sequence alignments. Bootstrap analyses of 1000 replications are carried out and bootstrap values > 50% are shown on the tree.

**Figure S3. Phylogenetic analysis of β-alanyl dopamine hydrolase (Tan) proteins.** The Tan-like sequences originated from Diptera species *Aedes aegypti*, *Anopheles darlingi* and *Drosophila melanogaster*; Coleopteran species *Henosepilachna vigintioctopunctata* and *Harmonia axyridis*; Hemiptera species *Nilaparvata lugens* and *Oncopeltus fasciatus*, and Lepidopteran species *Papilio machaon*, *Papilio xuthus*, *Papilio polytes*, *Biston betularia*, *Operophtera brumata*, *Bombyx mori* and *Chilo suppressalis*. The tree is constructed using the neighbor-joining method based on the full-length protein sequence alignments. Bootstrap analyses of 1000 replications are carried out and bootstrap values > 50% are shown on the tree.

**Figure S4. Depletion of *Hvadc* or *Hvebony* affects coloration in *H. vigintioctopunctata* adults.** The heads (A-C), pronotum (D-F), scutelli (G-I) and hindlegs (J-L) of adults whose third-instar larvae have received an injection of ds*egfp* or ds*adc* or *dsebony* were shown.

**Figure S5. RNAi of *Hvtan*** **had no significant influence on the cuticle pigmentation in larvae of *H. vigintioctopunctata*.** The newly-molted third-instar larvae were injected with an aliquot (0.1 μl) of PBS, or solution including 300 ng ds*egfp* or 300 ng ds*tan*. The larvae were then transferred to potato foliage. Two days and three days after injection, the mRNA level of *Hvtan* was measured (A). Relative transcripts are the ratios of relative copy numbers in treated individuals to those in ds*egfp*-injected larvae, which are set as 1. Different letters indicate significant difference at *P* value < 0.05 using analysis of variance with the Tukey-Kramer test. The dorsal and ventral views of the larvae had injected with ds*egfp* and ds*tan* (C) were shown. The heads (B) of the ds*egfp*- and ds*etan*-introduced larvae were further amplified. The unlabeled scale bars: 1mm.

**Figure S6. Silence of *Hvtan* had no significant influence on the cuticle pigmentation in adults of *H. vigintioctopunctata*.** The dorsal and ventral views of the pupae and adults from 3rd instar larvae injected with ds*egfp* (A, C, E, G) and ds*tan* (B, D, F, H) were shown. The elytra (I-L) were dissected from the adults whose 3rd instar larvae introduced with ds*egfp* and ds*tan*. The hindwings (M) and the color intensity (N) in equivalent regions of hindwings (circle 1 and 2 in H) were determined as mean gray values (average luminance) by *ImageJ* software. Data are shown as the mean values ± SE (n=6). NS indicates no significance in color intensity between control and test beetles (*p*>0.05, *t*-test). The unlabeled scale bars: 1mm.

**Figure S7. Depletion of *Hvtan* had no effect on the performance in *H. vigintioctopunctata*.** The newly-ecdysed third-instar larvae were injected with an aliquot (0.1 μl) of PBS, or solution including 300 ng ds*egfp* or 300 ng ds*tan*. The larvae were then transferred to potato foliage. The pupation and emergence rates were recorded during a 3-week trial period (A, B). The average values (± SE) followed by different letters indicate significant difference at *P* value < 0.05 using analysis of variance with the Tukey-Kramer test.
